# The Galaxy platform for accessible, reproducible and collaborative biomedical analyses: 2016 update

**DOI:** 10.1093/nar/gkw343

**Published:** 2016-05-02

**Authors:** Enis Afgan, Dannon Baker, Marius van den Beek, Daniel Blankenberg, Dave Bouvier, Martin Čech, John Chilton, Dave Clements, Nate Coraor, Carl Eberhard, Björn Grüning, Aysam Guerler, Jennifer Hillman-Jackson, Greg Von Kuster, Eric Rasche, Nicola Soranzo, Nitesh Turaga, James Taylor, Anton Nekrutenko, Jeremy Goecks

**Affiliations:** 1 Department of Biology, Johns Hopkins University, Baltimore, MD USA; 2 Institut de Biologie Paris-Seine, Université Pierre et Marie Curie, Paris, France; 3 Department of Biochemistry and Molecular Biology, Penn State University, University Park, PA, USA; 4 Department of Computer Science, Albert-Ludwigs-University, Freiburg, Freiburg, Germany; 5 Center for Biological Systems Analysis (ZBSA), University of Freiburg, Freiburg, Germany; 6 Academic Computing Services, Penn State University, University Park, PA, USA; 7 Department of Biochemistry and Biophysics, Texas A&M University, College Station, TX, USA; 8 The Genome Analysis Centre, Norwich, United Kingdom; 9 The Computational Biology Institute, George Washington University, Washington DC, USA

## Abstract

High-throughput data production technologies, particularly ‘next-generation’ DNA sequencing, have ushered in widespread and disruptive changes to biomedical research. Making sense of the large datasets produced by these technologies requires sophisticated statistical and computational methods, as well as substantial computational power. This has led to an acute crisis in life sciences, as researchers without informatics training attempt to perform computation-dependent analyses. Since 2005, the Galaxy project has worked to address this problem by providing a framework that makes advanced computational tools usable by non experts. Galaxy seeks to make data-intensive research more accessible, transparent and reproducible by providing a Web-based environment in which users can perform computational analyses and have all of the details automatically tracked for later inspection, publication, or reuse. In this report we highlight recently added features enabling biomedical analyses on a large scale.

## INTRODUCTION

Started in 2005, Galaxy (https://galaxyproject.org) enables biologists without programming and systems administration expertise to perform computational analysis through the Web (1). Existing analysis tools are defined for Galaxy and made available with a consistent Web interface. Multi-step analyses can be performed by running tools in succession, and Galaxy preserves the complete provenance of each analysis step (2,3). By bridging the gap between tool developers and researchers, Galaxy helps both constituencies accelerate their research. The project consists of several components:

**The Public Galaxy Server**: This is the focus of this paper. It is located at https://usegalaxy.org and is an installation (or instance) of the Galaxy software combined with many common tools, visualizations and data sources; this site has been available since 2007 for anyone to analyze their data free of charge. The site provides substantial CPU and disk space, making it possible to analyze large datasets. The public site supports thousands of users and hundreds of thousands of jobs per month (https://bit.ly/gxystats).

**The Galaxy software framework**: Galaxy is an open-source application (distributed under the terms of the permissive Academic Free License; https://getgalaxy.org) that can be deployed on any Unix system. The Galaxy software is highly customizable and integrates with a wide variety of compute environments ranging from laptop computers to clusters to compute clouds.

**The Galaxy Tool Shed**: The Tool Shed (https://usegalaxy.org/toolshed) facilitates sharing tools between instances of Galaxy by providing a central location where tool developers can upload both their tool configurations and ‘recipes’ describing how to install necessary dependencies. A Galaxy instance administrator can then install a tool and all necessary dependencies in an entirely automated way through the Galaxy Web interface or API.

**The Galaxy Community**: Finally, one of the most important components of the project is the Galaxy community. Galaxy has a broad community of users, tool developers and administrators who maintain Galaxy instances. Developments in the Galaxy community are described below in section *Community and Culture*.

Web-based genome analysis platforms are a long-term and growing area of research. Early platforms for doing genome analyses in a Web browser include Taverna (4,5), GenePattern (6) and Galaxy. More recently, iobio (7), Epiviz (8), the Genome Modeling System (9), and ZENBU (10) have been developed for performing genome analysis and visualization in a Web browser. Galaxy's large set of available tools, the powerful computing resources made available through the Public Galaxy Server and its large community set it apart from other analysis platforms.

### Goals of the Galaxy project

Three broad goals drive the development of Galaxy. First, Galaxy seeks to increase access to complex computational analyses for all scientists, including those with limited or no programming knowledge. Galaxy's Web-based graphical user interface (GUI) makes it simple to do everything needed for relatively large data analyses. Using the Galaxy GUI, users can upload their own data or retrieve data from public databases, choose analysis tools, set tool inputs and parameters and run tools. The Galaxy GUI also includes a workflow editor where users can create automated, multi-step analyses using drag and drop. Galaxy analyses are completely reproducible. All analysis parameters and inputs are permanently recorded, and analyses can be precisely repeated using the GUI. Finally, Galaxy provides collaborative and transparent analyses by enabling users to share and publish their analyses via the Web. Once shared, Galaxy analyses can be inspected at every level of detail as well as copied and extended.

Here, we provide an overview of the latest and most notable Galaxy features. These features impact nearly every aspect of the platform, from the core user interface to performance.

## CORE GALAXY FEATURES

### Analysis interface and history

**Figure 1.**
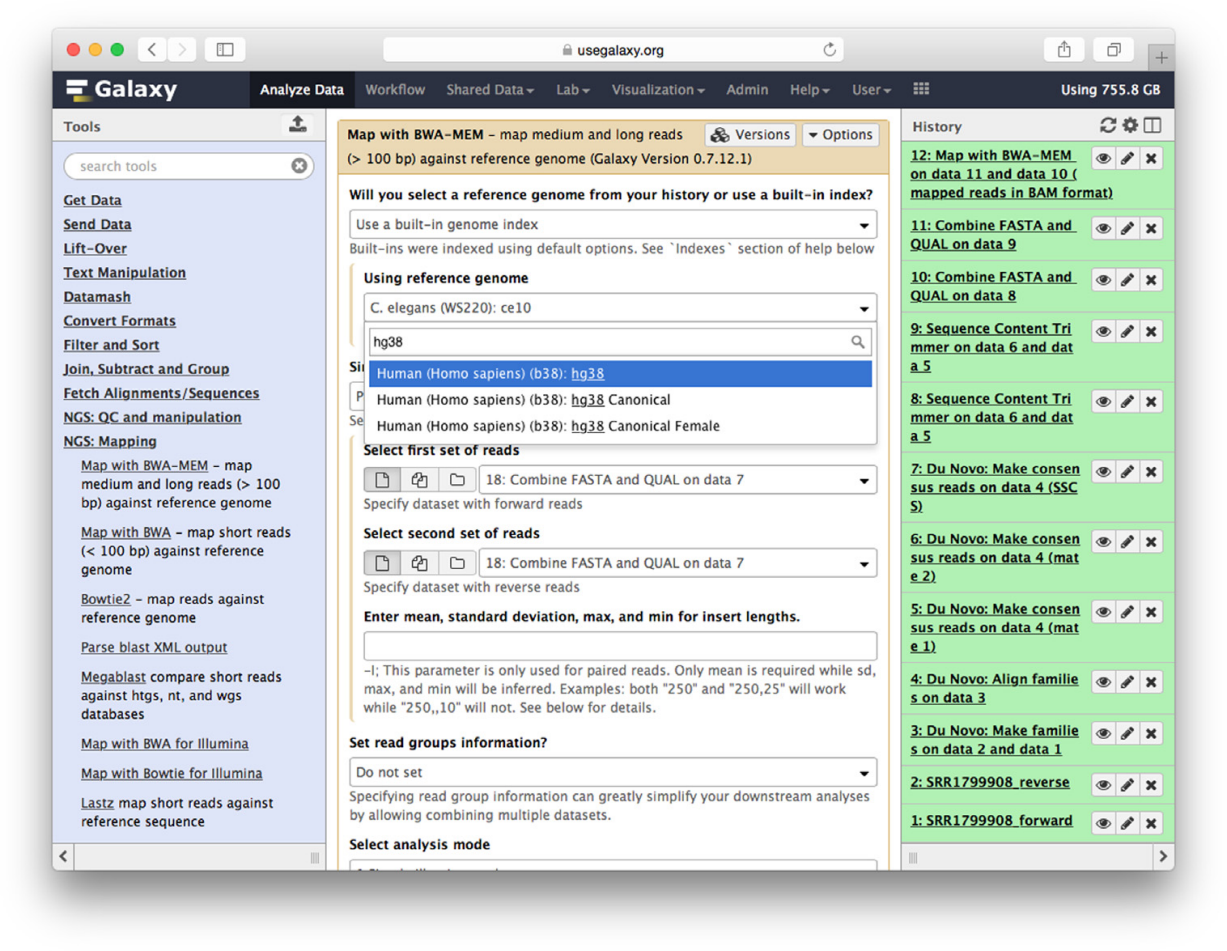
Galaxy analysis interface consisting of tool menu (left pane), tool interface (center pane), history (right pane).

The Galaxy analysis interface (Figure 1) is divided into three panels that show available tools, a main window for running tools and viewing datasets and a history of datasets created by running tools. Analysis tools are grouped into categories and can be searched. When a tool is selected, it is shown in the main window, where its inputs and parameters are set and the tool is executed. When a user executes a tool, its output datasets are added to the history panel. Color coding and animations show the state of tool execution. Clicking on a dataset in the history panel provides a wealth of information, including the tool and parameter settings used to create it, its size, and a small dataset preview.

### Workflows

**Figure 2.**
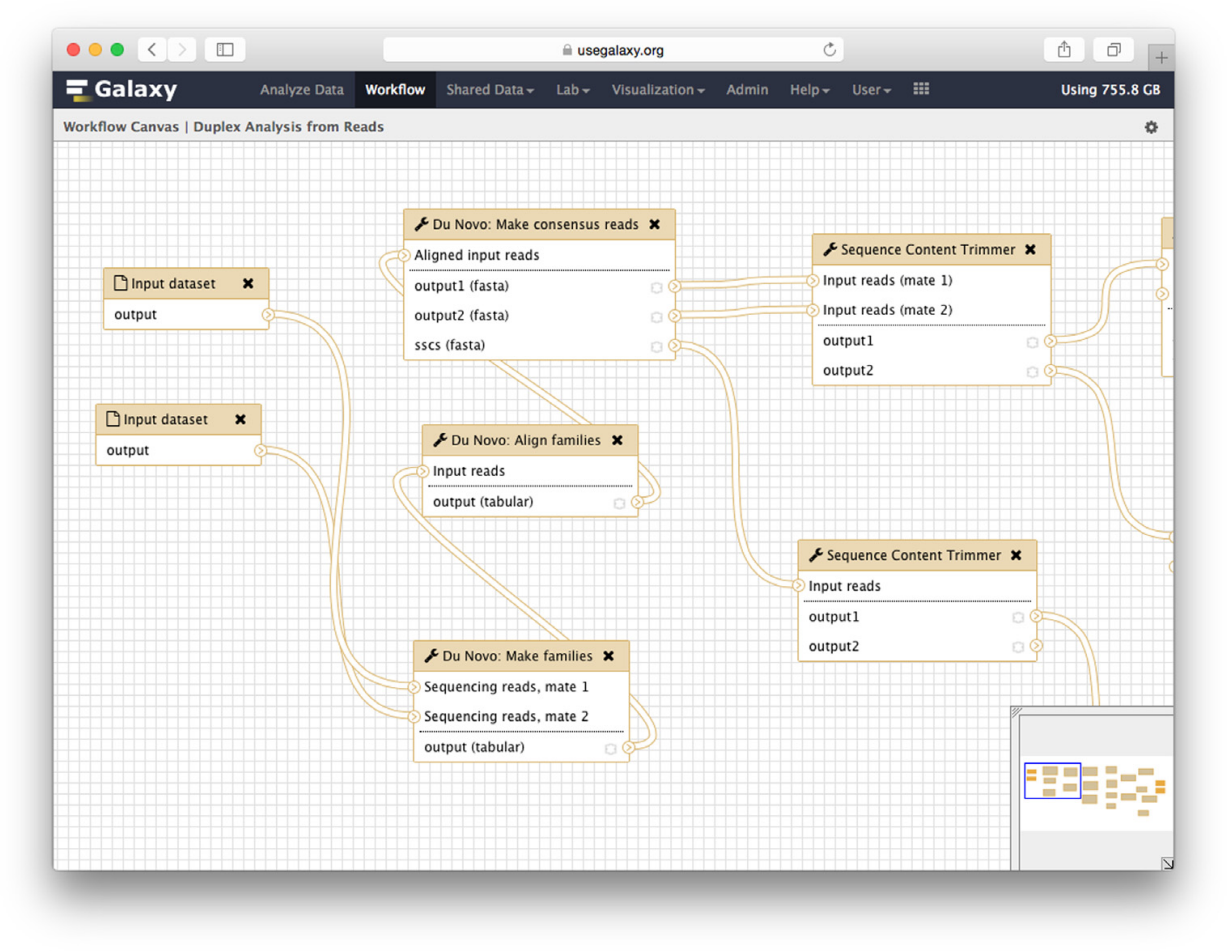
Galaxy's graphical workflow editor, show part of a sample workflow.

Galaxy's graphical workflow editor (Figure 2) makes it simple to create multi-step analyses using drag and drop functionality. Using the editor, tools can be added and connected to each other so that the output of one tool becomes the input of other tools. Tool parameters can be set, and common actions such as hiding intermediate datasets, changing data formats, and renaming datasets are supported as well. Workflows enable automating and repeatedly running large analyses. Once created, workflows function like tools: they can be accessed and run from Galaxy's main analysis interface.

### Sharing and publishing

Galaxy datasets, histories, workflows and visualizations can all be shared with individuals, or they can be shared via the Web using links such as https://usegalaxy.org/u/jeremy/h/hpac-exome-analysis Galaxy objects can also be published; published items are listed together and available to all users. Galaxy Pages are interactive web documents that include embedded and interactive Galaxy objects. Pages are often used for interactive methods supplements to papers and tutorials. Pages can be shared and published just like all other Galaxy objects.

## NEW GALAXY FEATURES

### Advances in analysis tools

#### Updated and large tool suite

Over the last year, we have increased the breadth and quality of tools available on the Public Galaxy server by adding 400 new tools to the server. More than 650 tools now enable users to analyze a wide variety of different genomic data. Very simple tools perform text manipulation and statistical operations, but the majority of tools on the server are for analysis of high-throughput genomic datasets from current DNA sequencers. High-throughput sequence analyses that can be run using Galaxy include quality control, genotyping and variant identification, motif finding, RNA-seq, ChIP-seq, and metagenomics. In addition to newly available tools, Galaxy recognizes and can process 300 well defined data formats (e.g. bam, bcf, bed, bedgraph) and offers visualization options and convenient conversion between different formats, as well as performance features like splitting inputs into small chunks and distributing them more efficiently over the computer cluster; we have also added EDAM (11) ontology identifiers.

We now use the Tool Shed (12) as the source for all new tools and version updates; the Tool Shed greatly simplifies tool deployment and ensures versioning and reproducibility. The number of Galaxy tools contributed by the community has greatly risen as a result of the Tool Shed. The Intergalactic Utilities Commission (IUC; https://wiki.galaxyproject.org/IUC), a volunteer community group, has emerged as the workhorse behind current tool development. Tool authors, including core Galaxy developers, can and often do submit tools to the IUC for review and approval via GitHub (https://github.com/galaxyproject/tools-iuc).

#### Tool user interface

All Galaxy tools use a single interface to simplify tool integration and maximize usability. In the past year, we have rewritten the tool interface to be significantly more responsive and dynamic, and we believe that these changes have substantially increased the interface's usability. We have recently extended this interface to include citations so that scientists can reference the methods used in their analysis. To ensure reproducibility, multiple versions of tools can be installed and users can run any installed version.

### Working with analysis histories

#### Multi-history viewer

**Figure 3.**
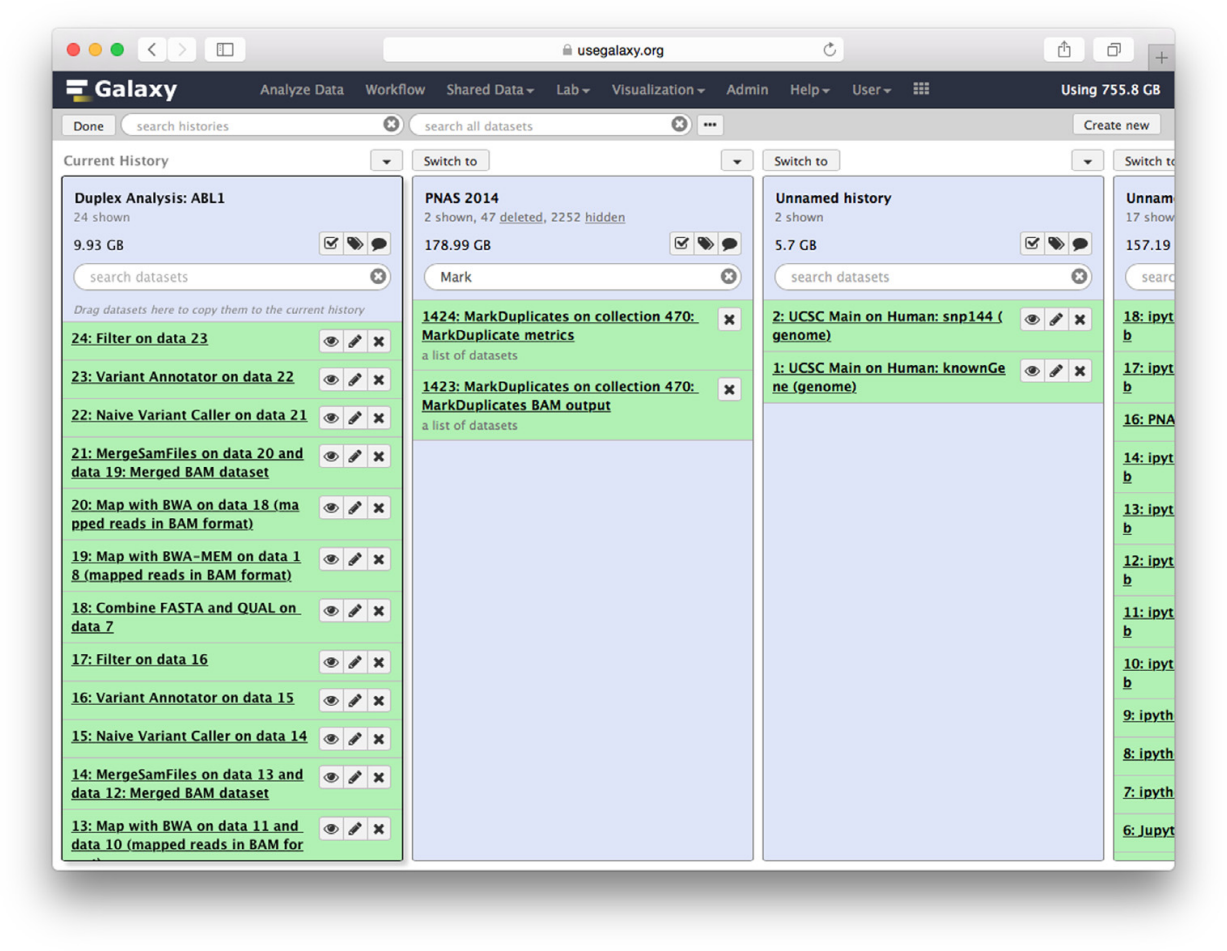
Multi-history viewer. Datasets can be copied among histories by dragging. Here one can also see the results of dynamic search functionality: in history **PNAS 2014** the search bar contains a partial keyword, Mark, causing the history to refresh and to show only datasets produced by the tool MarkDuplicates. As this particular history is very large (thousands of items) this functionality greatly simplifies analyses.

A new history management interface provides an intuitive and scalable way to manage histories and datasets (Figure 3). This interface enables all histories to be viewed together, allowing users to see the entirety of their work. Histories are shown with their complete list of datasets using the same interface as the main analysis page. All normal history and dataset operations can be used in this interface, such as annotating, tagging or deleting a history and searching or previewing datasets. Datasets can also be copied between histories by dragging and dropping.

#### History search and current state

Users can now search through their entire catalog of histories and datasets for partial text, data formats, references used, notes and tags (Figure 3). The current history, which can include thousands of datasets, can be searched by name, tag, or using metadata such as database = hg19. As a user searches, the history dynamically updates to show only matching datasets. When a user selects a dataset for editing, display, or viewing analysis details, the dataset is now highlighted in the history panel. This allows a user to easily track the dataset currently in use.

### Manipulating, storing and sharing genomic data

#### Uploading data to Galaxy

Using our new interface for uploading data, users can simultaneously upload an unlimited number of files to Galaxy. Upload can be started, paused and reconfigured at any time, and its progress is always visible. Files can be added via drag & drop from a local computer, selected from files uploaded via FTP, or uploaded by URL.

### Public genomic data

#### Reference data

Reference data, especially reference genome sequences, indices, and gene annotations, are needed for many Galaxy tools. Our Public server now has over 175 reference genomes, and we add 5–10 new genomes every 6 months. Genomes are obtained from public data repositories, including NCBI (13) and UCSC (14). Galaxy includes a custom genome feature for researchers working with genomes that are not available from these sources. This feature enables researchers to use Galaxy tools with their own genomes or transcriptomes.

We recently transitioned from a manual process to build reference data to an automated process using Data Managers (15). This transition greatly increases the ability to track provenance and the reproducibility of these critical resources. We have also implemented a Data Manager for Galaxy administrators to fetch and install reference data that are available on our public server. This data manager turns our public server into a large reference data repository that can be used to populate reference data in local Galaxy servers.

#### Data libraries

Galaxy data libraries provide a convenient method to share data with collaborators or the general public. We have recently developed a dynamic client-side user interface for working with data libraries that is, on average, five times faster than the previous server-side interface. While the previous interface would not work for libraries with tens of thousands of items, this interface can be used for browsing these very large libraries. We also continue to expand the datasets in our libraries on the main Galaxy server: there are now more than 2000 datasets publicly available.

### Scaling to large analyses

#### Dataset collections

**Figure 4.**
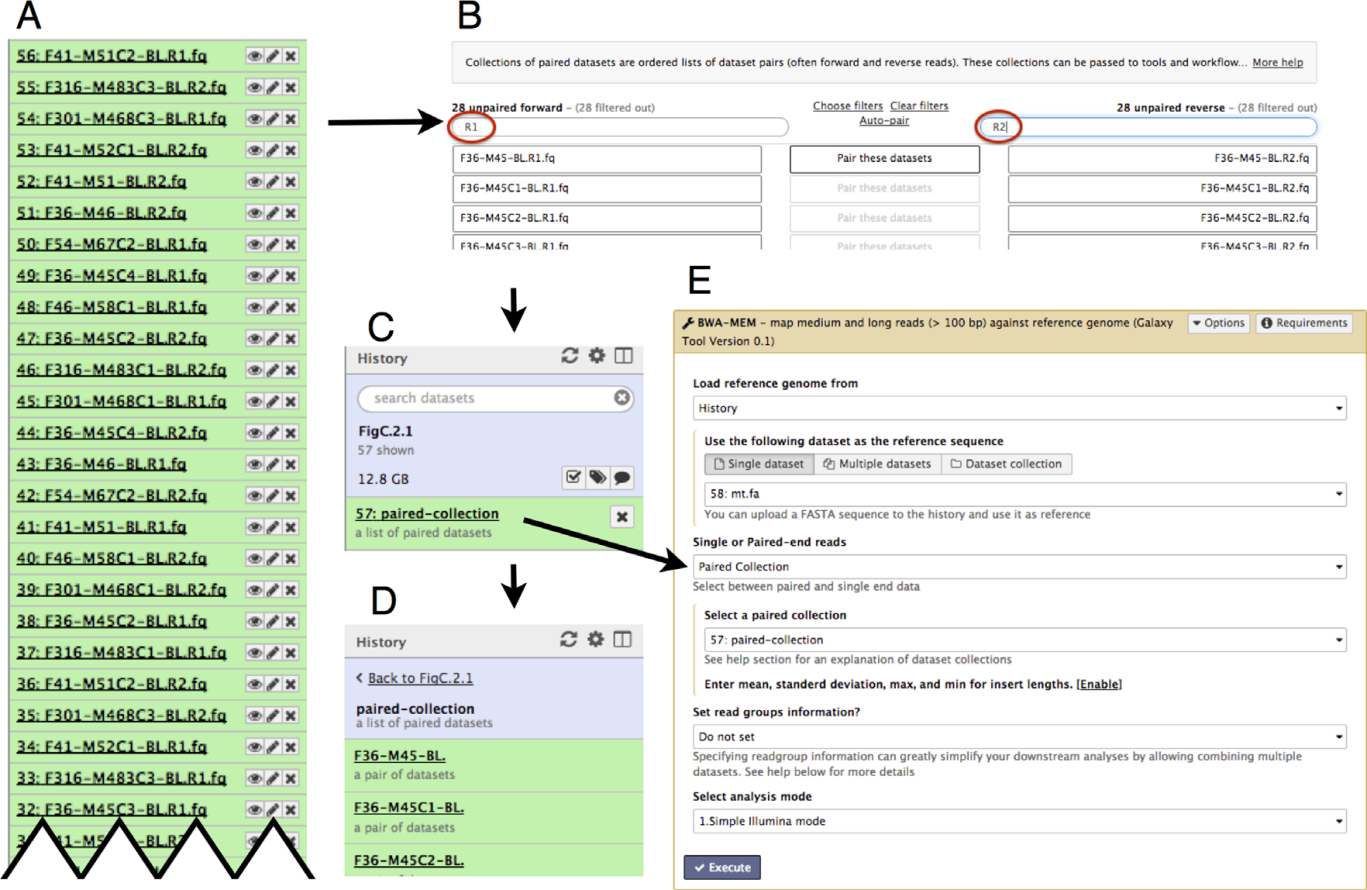
(**A**) Dataset collections simplify analysis of large numbers of files. A Galaxy history with a paired-end DNA re-sequencing dataset from 28 individuals contains 56 files (each green box is a file). It is difficult to understand this history because there are so many files and because forward (R1) and reverse (R2) reads are unordered. As these files are analyzed and more outputs/files are created, it becomes very difficult to navigate around the history and understand how files are connected as inputs and outputs of particular tools or analyses. Dataset collections make analysis of this mix of files straightforward by grouping all files into a collection that can be analyzed as a single unit. This example demonstrates using collections with paired end data, but collections can be created for any set of files. (**B**) Creation of a paired collection from the history shown in panel A. Because dataset names use a uniform nomenclature for forward and reverse reads, the collection creation form can automatically determine pairings. (**C**) Pairing these datasets generates a single item (a **Collection**) in Galaxy's history. (**D**) Clicking on this newly created Collection expands it and shows its content (only first three datasets are shown). (**E**) Galaxy's BWA interface takes the entire dataset collection as a single input.

Today it is common to analyze multiple datasets derived from different individuals, conditions, treatments, environmental sites or tissues types. To enable analysis of many datasets simultaneously, we have developed Galaxy dataset collections (Figure 4). A collection contains related datasets and defines their relationships. Galaxy currently supports two types of collections: a simple collection of multiple datasets and paired collection containing dataset pairs, such as those generated by paired-end or mate-pair sequencing. For Galaxy users, collections can be used just as single datasets are used. When a user executes a read alignment tool on a collection of 100 paired-end datasets, the tool will run 100 times to align each dataset pair.

#### Improved workflows

Galaxy workflows have been extended in several important ways. Galaxy now tracks the state of a workflow throughout its evaluations so that workflows can be paused and restarted. Dataset collections are fully compatible with Galaxy workflows as well, and workflow steps can either iteratively process a collection or combine a collection of datasets into a single resulting dataset. Galaxy workflows can now be embedded in other workflows and used as a single step in the parent workflow, just like any other tool. Embedded workflows make it much easier to create and manage complex analyses in Galaxy's graphical workflow editor.

### Cloud Launch: moving analyses to the cloud

Despite the sizable computing resources available on the main Galaxy server, there are limitations to ensure fair and safe access for everyone. First, there are quotas on the amount of data users can have and the number of concurrent jobs a user can run. Second, users cannot add their own tools to the server. One way to avoid these constraints is to use cloud computing resources to rent necessary computing and storage resources. To support this we have made the entire Galaxy platform available on the Amazon cloud (16).

Using https://launch.usegalaxy.org, users can start their own Galaxy server that they control every aspect of. Personal Galaxy cloud servers come preconfigured with over a hundred tools and hundreds of gigabytes of reference genomic data. Each instance is entirely customizable by a user allowing them to install additional tools and data, and there are no storage quotas. To start a Galaxy cloud server, a user supplies her cloud access credentials, a cluster name and chooses the type of hardware to use. Once started, the entire Galaxy platform is quickly available. Once the need for the launched Galaxy instance subsides, the user can shut down their cluster and return to it at a later time, with all the data having been preserved. Over 2,400 Galaxy cloud servers were launched in 2015.

### Training materials and community support

The Galaxy Project has introduced a wiki Website, mailing lists, and screencasts to help the thousands of users that want to learn how to use Galaxy. However, as usage has grown, these approaches have become inadequate. To address this challenge, we have developed new training materials that can scale to support increasing numbers of Galaxy users.

#### Interactive tours

Interactive Tours provide an interactive and self-paced approach for learning how to use Galaxy. Each tour is a series of steps that highlight a particular user interface element and ask a user to use the element to perform a small task, such as opening a window, changing a parameter or running a tool/workflow. There are several tours on the Galaxy server, including tours showing how to run a workflow, how to share an analysis history and how to complete a basic RNA-seq analysis. Tours run completely within Galaxy and can include their own sample datasets.

#### Analysis exercises

We have developed Galaxy Pages that provide hands-on exercises for scientists to learn about how to use Galaxy for a variety of analyses. Exercises for ChIP-seq, RNA-seq, variant calling, and metagenomics provide sample datasets, descriptions of each analysis step and questions to answer as the analysis is completed. These exercises have been used as assignments in university bioinformatics courses (https://wiki.galaxyproject.org/UniversityCourses) and in Galaxy training workshops (https://wiki.galaxyproject.org/Events).

#### Community knowledge portal

We have created a custom BioStar (17) question & answer Website to respond to and archive Galaxy user questions (https://biostar.usegalaxy.org/). In BioStar, users ask questions and other users can provide answers, which are then voted on by other users, and can be chosen by the inquirer as the approved answers. In contrast to a classic time-ordered forum, this allows the ‘correct’ or most useful answers to rise to the top. By leveraging gamification (18) through the use of ‘reputation’ points, users are encouraged to participate. Our BioStar site runs parallel to the primary BioStar site (which is for general bioinformatics questions), provides seamless log-in integration with the Galaxy server, and allows users to post questions directly from within Galaxy Tools. Researchers using Galaxy can come together and share both scientific advice and practical tool help. Although BioStar has entirely replaced our galaxy-user mailing-list, users can enable ‘mailing list mode’ to send emails on every user post.

### New visualizations

**Figure 5.**
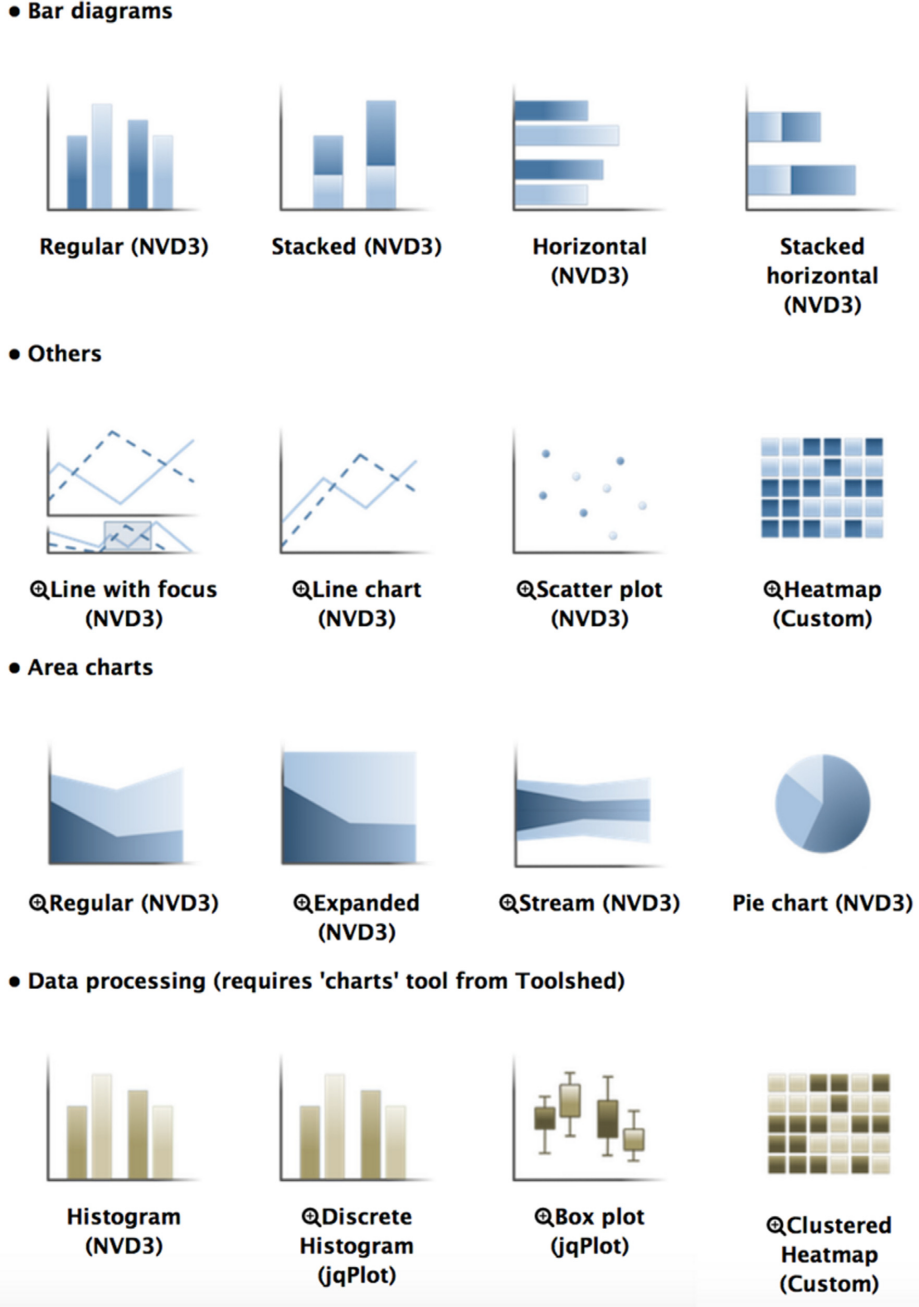
Selection panel for Galaxy numerical visualizations showing the variety of plots that can be created.

Leveraging Galaxy's visualization framework (19), there are now more than 20 visualizations available in Galaxy. Visualizations include a genome browser, a Circos plot, a phylogenetic tree viewer and many numerical visualizations (Figure 5). These visualizations use Galaxy to simplify data visualization by automatically and transparently preprocessing data, such as transforming data formats, indexing data and aggregating data. With the addition of these visualizations, we have made strides toward making Galaxy a complete analysis environment that supports everything from data processing to interpretation.

### Community and culture

The Galaxy community is a vital component of the project. We have taken several steps to strengthen and grow in the Galaxy community in recent years. The Galaxy Training Network (https://wiki.galaxyproject.org/Teach/GTN) highlights training materials and workshops hosted by community members. We have also expanded opportunities for the community to gather online and in person. The GalaxyAdmins group meets online every other month, hackathons and regional Galaxy events (both virtual and in-person) happen throughout the year and around the world, and the yearly Galaxy Community Conference gathers over 200 community members for 5 days of training, hacking, presenting and networking (see https://bit.ly/gxy-events).

Galaxy is an open-source project (https://github.com/galaxyproject/galaxy), and in recent years we have taken several steps to make project participation more open and inclusive. We introduced a formal process for project procedures and expanded the core development team to include members from seven different institutions. Through these processes and increased diversity, we now provide a transparent and documented way to help new developers start contributing to Galaxy and to enable strong contributors to become core team members.

We have also implemented approaches to improve code quality, including a formal code review process for all proposed modifications. We use two continuous integration services (Travis CI [https://travis-ci.org/] and Jenkins [https://wiki.jenkins-ci.org/]) which run a series of tests including code conformance, unit tests, functional tests, application programming interface (API) tests on each new pull request, effectively preventing most software regressions.
